# The Transport and Outcome of Sick Outborn Neonates Admitted to a Regional and District Hospital in the Upper West Region of Ghana: A Cross-Sectional Study

**DOI:** 10.3390/children7030022

**Published:** 2020-03-20

**Authors:** Edem M. A. Tette, Benjamin D. Nuertey, Dominic Akaateba, Naa Barnabas Gandau

**Affiliations:** 1 Department of Community Health, University of Ghana Medical School, P.O. Box 4236, Accra, Ghana; ben.nuertey@gmail.com; 2 Public Health Department, Tamale Teaching Hospital, P.O. Box TL 16, Tamale, Ghana; 3 Upper West Regional Hospital, P.O. Box 6, Wa, Ghana; doeakansiadi1@gmail.com (D.A.); naabarnabas@gmail.com (N.B.G.); 4 School of Medical Science, University for Development Studies, P.O. Box TL 1350, Tamale, Ghana

**Keywords:** neonatal, transport, travel, distance, hypothermia, mortality, newborn

## Abstract

Optimum care of sick neonates often involves transporting them across different levels of care. Since their condition may deteriorate over time, attention needs to be paid to travel distances and how they are transferred. We examined the mode of transport, distances travelled, condition on arrival and outcome of outborn neonates admitted to a district and a regional hospital in Ghana using a cross-sectional study involving caregivers of neonates admitted to these hospitals. Information on referral characteristics and outcome were obtained from questionnaires and the child’s case notes. Overall, 153 caregivers and babies were studied. Twelve deaths, 7.8%, occurred. Neonates who died spent a median duration of 120 min at the first health facility they visited compared with 30 min spent by survivors; they travelled mostly by public buses, (41.7%), compared with 36.0% of survivors who used taxis. Majority of survivors, 70.2%, had normal heart rates on arrival compared with only 41.7% of neonates who died; hypothermia was present in 66.7% compared with 47.6% of survivors. These findings indicate that the logistics for neonatal transport were inadequate to keep the neonates stable during the transfer process, thus many of them were compromised especially those who died. Further studies are warranted.

## 1. Introduction

Child mortality is largely due to deaths occurring in the newborn period or the first 28 days of life [1]. In 2015, global neonatal deaths accounted for 45% of deaths in children under the age of five years and are projected to increase to 52% by 2030 [2]. Accordingly, considerable emphasis has been placed on improving facility-based care for newborns [3,4,5]. Patient outcomes are also known to be related to the distance patients travel to access care. Several studies have shown that outcomes worsen with increasing distance from a patient’s residence [6,7]. Neonates are particularly vulnerable to the distances they have to travel to receive care as their condition often deteriorates rapidly [6,8,9]. Thus it is imperative that neonates receive the necessary specialised care as quickly and as close to their residence as possible [6,9]. A systematic review of 40 studies on neonatal transport in developing countries found that 11 studies had reported hypothermia as an important risk factor for morbidity and mortality [10]. Other factors which influenced outcome were hyperthermia, hypoglycaemia, poor perfusion and duration of transport. They also found that, the capacity to provide additional condition specific care prior to and during transfers for neonates with prematurity and respiratory distress, surgical conditions and complicated sepsis also impacted on outcome. Family members accompanied most of the transfers. However, some studies showed that using specially trained transport teams was associated with better physiological stability whether the journey was long or short. A study conducted in rural North-Western Ethiopia also demonstrated the importance of travel time by showing that children under the age of five years who lived 1.5 h or more from a health centre had a two to three times higher risk of mortality than those of who lived less than 1.5 h from the health centre [11]. A study from Burkina Faso on children under the age of five years found a median walking time of 1 h to the closest health facility. However, it was six and eight hours during the dry season and rainy season, respectively, to reach a health facility and mortality risk was 50% higher after a walking distance of 4 h [12].

A multiple variable logistic regression and meta-analyses model used in pooled data of 29 demographic and health surveys (DHS) from 21 low and middle income countries between 1990 and 2011 consisting of 124,719 mothers and 126,835 births showed that the odds of neonatal mortality increased with distance [13]. It was higher at 7.7%, 16.3% and 25% for children living 2, 3, and 5 km, respectively, from a health centre compared with those who lived within 1.0 km from a facility. For distances 10 km or more, the neonatal mortality risk was 26.6%. They found that children from households located more than 60 min from health facility were at 25.6% risk of neonatal deaths than children living within 10 min from a health facility. They reported that distances to facilities were not only relevant when they are far, but even small distances have sizable increases in health service utilisation and effect on neonatal mortality. They also noted that pooled effects of this study provided a large sample size and sufficient power to detect the effects of distance on child mortality which has been a major challenge for other studies with smaller sample sizes [14].

In the developed world, improvements in neonatal outcomes have partly been attributed to the regionalisation of neonatal care [15]. This involves the organisation of care and transfer of high-risk pregnancies and neonates across three different levels of care. Simultaneous attention is paid to the transport of the sick newborns using specially trained transport teams and mechanisms ranging from ambulances with transport incubators to helicopters in order to ensure that their condition does not deteriorate during transfers [15,16]. Unfortunately, limited attention is paid to the distance between facilities and the transport of sick newborns in the developing world as many newborns arrive at the referral facility in a cold and undesirable state, while others do not complete the referral process at all due to access, distance, mode of transport, cost and other logistic barriers [9,17,18,19,20]. In most places, the outcome of babies born or referred from outside a hospital is often worse than those referred immediately after delivery to a neonatal unit within the hospital in which they were delivered [21,22]. The Upper West Region in Ghana has the most rural population and according to the Regional Health Directorate, the region has the least amount of tarred roads per kilometre compared to the other regions [23,24]. Only two of the nine administrative district capitals in the region are reported to be linked to each other and to the regional capital by tarred roads [24]. Thus many of the roads are untarred and during the rainy season, navigating these roads can be challenging. The main means of transport is by cars, buses, bicycles, tricycles, and motorcycles. This study examined travel time, distance, mode of transport and condition on arrival of outborn neonates who died and survived at the Upper West Regional Hospital (UWRH) and St Joseph’s Hospital, Jirapa, (SJH).

## 2. Materials and Method

### 2.1. Study Location

The Upper West Regional Hospital is a 200-bed hospital which acts as a regional and municipal hospital located at Wa, the regional capital. The hospital serves the Upper West Region and has 9 wards, including a neonatal unit which was established in August 2016. The number of deliveries conducted there from January to December 2016 was 4915. St. Joseph’s hospital in Jirapa functions as a district hospital for the people of Jirapa, which is also located in the Upper West Region of Ghana. The hospital has 7 wards including a neonatal unit which was established in 2015. The number of deliveries in the year 2016 was 1709. The hospital attends to referrals from health centres, Community-Based Health Planning and Services (CHPS) compounds and some of the other district hospitals in the region. 

### 2.2. Study Design

The study was a descriptive cross sectional study which was part of a larger study of neonatal mortality in the Upper West Region. The focus of this part of the study was to examine the distance travelled, travel time and mode of transport available to outborn patients referred to both hospitals who died and survived. The primary outcome was neonatal mortality. However we also described pertinent clinical factors associated with neonatal transport [10]. This included the clinical condition of the neonates on arrival at these health facilities and their diagnoses to provide a better understanding of the context of this study and to identify patients who are likely to benefit from better case management before or during transportation [10].

### 2.3. Sampling and Sample Size

A sample of convenience was used. Parents or caregivers of consecutive outborn neonates admitted to both facilities from outside these hospitals were recruited and interviewed by research assistants on weekdays from Monday to Friday. Caregivers who were unavailable, such as those whose babies did not survive and/or had left the hospital before they could be interviewed and could not be captured at a follow-up visit, were excluded.

### 2.4. Study Population

Parents or caregivers of newborns admitted to the neonatal unit who delivered outside these hospitals in 2018 were eligible for the study. Parents or caregivers who were unavailable to provide accurate information on the referral process were excluded. Outborn newborns admitted to the Upper West Regional Hospital (UWRH) over an 8 month period from 23 January to 16 October 2018 and St Joseph’s Hospital (SJH) from 23 January to 31 June 2018 were studied.

### 2.5. Data Collection

Data on neonatal referral characteristics was collected on outborn neonates referred to and admitted from UWRH and SJH. This was accomplished by administering questionnaires to mothers or caregivers and reviewing the child’s case notes. Information including the child’s residence, the place the child was referred from, transportation details, sources of delay and the time between referral and presentation were collected. Information was also collected on the condition of each neonate on arrival. This included the respiratory rate, heart rate and temperature as recorded in the child’s case notes. This was later classified as normal or abnormal using reference values [25,26]. Demographic features such as age, sex and clinical characteristics, such as presenting features and outcome of the neonate, were also obtained.

### 2.6. Data Analysis

The data capture and analysis was done using Statistical Package for Social Sciences (SPSS) version 16.0. A summary of the data was done using frequencies, proportions, means, medians, standard deviation and inter-quartile ranges of study variables. These were mostly presented in tabular form. A GIS map showing the location of the study, residence of patients and health facilities was also presented. The Chi square test was used to compare patient characteristics at baseline among those who survived and those who died. A probability level of 5%, that is, a *p*-value of less than 0.05, was the accepted level of statistical significance.

### 2.7. Ethical Clearance

The Ghana Health Service Ethical Review Committee provided the ethical clearance to conduct this study—(Ethical Review committee Protocol ID No: GHS-ERC 09/03/17). Consent was obtained from participants before the questionnaires were administered. Permission was obtained from the management of the two hospitals. The data was anonymized to conceal patient’s identity; the analysis was conducted in a way that would not link the final results to individual patients.

## 3. Results

### 3.1. Patient Characteristics

Altogether data were obtained from 153 caregivers of outborn neonates referred to both hospitals, with 140 (91.5%) from the UWRH and 13 (8.5%) from SJH. Mothers were the caregivers of 145 of these babies. In all, 14 (9.2%) of patients were less than 24 h old, whereas 62 (40.5) were one to seven days old and 77 (50.3%) were more than a week old. The median age was seven days with interquartile range of two to nine days. There were more male (88, 57.5%) than female (65, 42.5%) patients. Preterm babies formed 26 (17.0%) of the neonates. Twenty-one babies (13.7%) were low birth weight. No babies weighed < 1000 g and only one baby had a weight between 1000 to 1499 g, (0.7%). Table 1 shows the characteristics of participants. 

### 3.2. Distance Travelled

**Figure 1:**
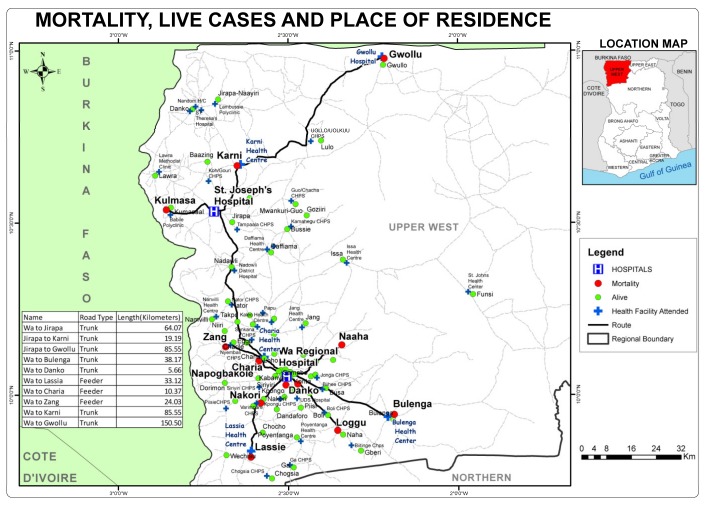
Mortality, live cases, place of residence and health facilities visited by outborn neonates.

The median distance travelled was 21.17 km (range 2.69–75.66 km) for those that died and 18.27 km (range 0.93–91.15 km) among the survivors. The proportion of neonates who travelled a distance of more than 4 km to reach the referral hospital among those who died was 91.7% versus 84.3% for survivors. For a distance greater than 8 km, the proportions were 83.3% for those died and 76.4% for survivors. Eight out of the 12 (66.7%) babies who died travelled approximately 12 km or more. Altogether, 43 (28.3%) babies travelled < 10.00 km, 40 (26.3%) travelled 10.00–19.99 km, 65 (42.8%) travelled 20.00–79.99 km and 4 (2.6%) travelled ≥ 80 km. The overall mean was 23.1 km (SD 20.1). Figure 1 is a GIS map showing the place of residence of the 12 patients who died, the residences of 140 survivors and the location of health facilities visited. 

### 3.3. Mode of Transport

Table 2 provided information on mode of transporting sick neonates to the referral hospitals. Taxi was the most common means of transport used by about a third (52, 34.0%) of patients. This was followed by the use of a bus by 45 (29.4%) and motor cycles for transport by over a fourth of patients (41, 26.8%). The child’s mother accompanied the child in the majority of cases (145, 94.7%) and the father was in attendance in 68 (54.8%) of cases among those accompanied, while other family members were in attendance in about a third of cases (40, 32.3%). No significant association was found between mode of travel and outcome.

### 3.4. Travel Time

**Figure 2:**
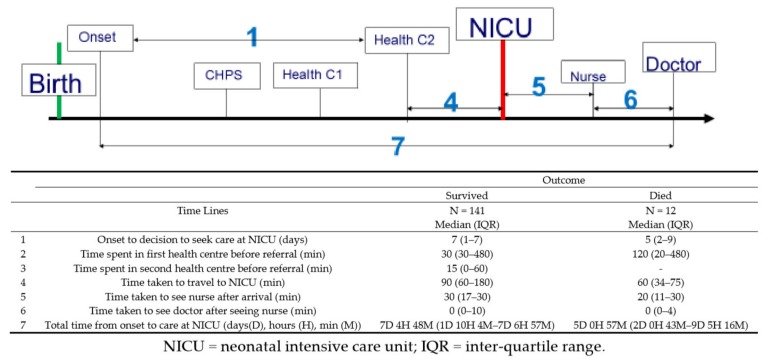
Timeline analysis of referral pathway of outborn neonates referred to the neonatal unit.

The median time spent travelling to the referral facility was 1 h 20 min (interquartile range, 1–3 h), as shown in Figure 2, and it ranged from a minimum of 15 min to 48 h (two days). The mean time it took to see the doctor on arrival at the referral facility was 37.85 min (SD 67.28 min), the median was 30 min and it ranged from 0 min to 12 h. Table 2 and Figure 2 shows the travel time and sources of delay as described by caregivers for neonates stratified by survived or dead outcome.

### 3.5. Outcome

Our primary outcome was mortality so we compared patients who died with those who survived. Hypothermia (temperature below 36.5 °C) was present in 75 (49.0%) on arrival. Fever (temperature > 37.4 °C) occurred in 60 (39.2%). There were 117 (76.4%) neonates with at least an abnormal heart rate (heart rate < 110 or >160 beats per min), respiratory rate (respiratory rate of <30 or >60 breaths per min) or moderate to severe hypothermia (temperature 34–35.5 °C) who were considered unstable. This is highlighted in Table 3, which provides a summary of the condition of the neonates on arrival and their main diagnoses according to outcome. In all, 12 (7.8%) of the babies died, comprising three males and nine females. The neonates who died spent a median of 120 min (20-480 IQR) at the first health facility, compared with a median of 30 min (30–480 IQR) spent by neonates who survived. Regarding the mode of transport, 41.7% of those who died, travelled to the hospital by public transport (bus), whereas, a majority of those who survived 36.0% (50) came to the hospital in a taxi. Altogether 70.2% (99) of those who survived had normal heart rate (110 to 160 beats per min), on the other hand only 41.7% (5) of those who died had normal heart rate. In addition hypothermia was present on arrival in 66.7% (8) of those who died compared with 47.6% (67) of those who survived. In all, 91.7% of those who died arrived in an unstable condition compared with 75.2% of those who survived. Only one of the babies who died did not have any of these abnormal clinical signs.

## 4. Discussion

The referral of sick neonates to tertiary hospitals is inevitable as a proportion of neonates will require specialized medical or surgical care [20,27,28,29]. This poses many challenges including a financial burden on families, long travel distances which may lead to incomplete referrals and poor outcomes [19,20,29]. In this study, the median time it took to arrive at the neonatal units was 60 min for neonates who died and 90 min for those who survived; however, there were outliers. One patient took 48 h to complete the journey to the neonatal unit. In all, eight (66.7%) of the babies who died travelled approximately 12 km or more. Bad roads were reported by some caregivers, most likely due to the rural nature of the region and poor development of the road infrastructure [24]. For each baby who made it to the hospital, there may be several others who did not attempt the journey at all or make it all the way [19]. Studies from Ghana and elsewhere show that proximity to a health facility generally improves service utilisation and outcome as long distances delay access to emergency care [6,7]. A study at the teaching hospital at Ibadan, Nigeria reported that 19 babies out of the 401 neonates they studied were brought in dead on arrival [30]. A study from Bangladesh also found that travel distances of more than 3 h were associated with increased risk of mortality [31].

In this study, hypothermia was common, occurring in almost half, 49.0%, of all the babies, but it occurred in 47.6% (67) of those who survived compared to 66.7% (two-thirds) of the babies who died. The proportion of hypothermia is lower than that of a study in India which reported 76% [17]. However it is worse than a study of 73 babies in Cameron which reported hypothermia among 20% and a study of 150 neonates in Bangladesh, which reported 14% [9,32]. While hypothermia may be associated with medical conditions such as prematurity and inadequate stabilisation of these babies before transfer, it might have been aggravated by long travel distances to reach the destination hospitals, the mode of transportation and the form of thermal protection (e.g., wrapping or kangaroo mother care used during the journey) [20,26,27,29,30,33]. Our patients might have been affected by some of these factors. In recent times, devices for transporting newborns across facilities such as the Embrace have been designed specifically for use in developing countries to keep babies warm during the transfer process [34]. Much of the guidance on newborn care rightly places emphasis on keeping babies warm; however, while thermal protection is important it is worth noting that fever was present in 40.5% of the neonates. Thus, it is equally important to avoid overheating babies and to control those with fever due to neonatal sepsis, malaria or other infections [35,36]. Since this is one of the hottest regions in the country, further studies are needed to evaluate the advice given during transfers to see if it is appropriate. We also noted abnormal breathing patterns in 62.5% of the neonates but there were similar proportions of tachypnoea or fast breathing among neonates who died and the survivors even though we used the highest upper limit of normal ranges [25].

The majority of neonates who survived, 70.2% (99), had a heart rate within normal range on arrival, compared with 41.7% (5) of those who died using 110 to 160 beats per min as normal heart rate [25,37]. Though there are some controversies about what is normal, heart rate is reported to be a vital indicator of health in a neonate and can be used to predict infection [38,39,40]. While a high heart rate may be an indicator of infection, a low rate usually indicates impending respiratory or cardiac failure and the need for resuscitation [37,38,39]. A study of 50 neonates transferred to a tertiary facility in Jamaica found that 28% of the neonates needed cardiopulmonary resuscitation on arrival [41]. Half of these neonates experienced cardiopulmonary deterioration during the journey and 61% of the neonates who died required, cardiopulmonary resuscitation on arrival. This implies that heart rate must be stable before and monitored during transfer. Thus, the Turkish Neonatal Society guideline on the safe transport of newborns includes a heart rate ranging between 120–160 beats per min, in their stabilisation criteria before leaving hospital, for neonatal transfers [42]. 

The commonest mode of transport was by taxi (cab) followed by bus, tricycle and motor bike, but for babies who died, public transport (Bus) was the commonest mode of transport, used by 41.7%. These forms of transportation were also used in studies from India, Uganda, Nigeria and Bangladesh [17,21,30,31,43]. One would have expected the use of motorcycles in particular to expose these babies to strong wind currents and increase the risk of hypothermia. However, in this study, we found no significant association between mode of travel and outcome. Travelling by ambulance is the ideal, but only one neonate travelled by ambulance in this study. Similarly, a low use of 4% was reported in Nigeria [30]. Our finding is much lower than 11% (108 ambulances) reported in India [17], the 22.7% reported by a tertiary facility in India [43] and the 87% (130) reported in Bangladesh [32]. It is lower than the ambulance transfers done in 71.3% of neonates who died and 68.2% of those who survived in another Bangladeshi study [31]. It also seems lower than a South African study which described 120 road ambulance transfers occurring in one district over a year and an Indian study which reported that ambulance transfers formed 75% of inter-facility transfers [44,45]. Our findings may be a reflection of the low level of resources available in this region. Travelling by ambulance usually provides a source of oxygen, equipment for resuscitating newborns, drugs and other supplies to stabilise the infant during travel [28,33,44].

Even when the transport infrastructure is ideal, it is important that neonates are accompanied by trained health professionals carrying additional equipment or drugs to deal with any untoward events that may occur on the way [20,28,32,33,44]. These professionals also support the caregiver and provide additional information to the destination hospitals when necessary. In this study only one patient was accompanied by a trained professional, a nurse. This is low but similar to a study from Bangladesh which revealed that only six (4%) of the 130 ambulance trips were accompanied by any medical personnel [32]. However, a much higher percentage (40%) was reported in a study from India [43]. Mothers in this study were most commonly accompanied by family members followed by friends who would also have provided some emotional support. 

Our findings of a mean distance of 23.1 km is twice as long as that of a study done at the University College Hospital (UCH), Ibadan, in an urban setting, which showed that outborn neonates admitted to the hospital travelled a mean distance of 10 km and it ranged between 0.5 km to 80 km [30]. In addition, while the majority of the outborn neonates (61%) came from within a 10 km radius of UCH, the majority of patients in this study (72%) travelled 10 km or more, with 45.4% of the patients travelling 20 km or more compared with only 2% of patients from UCH. Thus, according to the study involving 126,835 births, logistic regression and meta-analysis of 29 DHS data, these children would be at least at 26.6% higher odds of neonatal mortality [13]. Geographical isolation of communities from health facilities in this region has been reported to be a barrier to neonatal care, compounded by the lack of ambulances and a poor road network making inter-facility travel distances longer than they need to be [46].

We found that there were more female, premature and low birth weight neonates among the babies who died and the differences between those who died and those who survived were significant. The finding of more premature and low birth weight infants among the babies who died is not unexpected, as this has been found in other studies including studies on outborn babies [1,21,30]. What is different is that neonatal mortality is usually greater in male babies [1,30]. Thus finding greater mortality among females may be related to differences in care seeking behaviour for the sexes but this must be interpreted with caution given the limited numbers of patients. Other details on sex differences and socio-cultural practices in the neonatal experience of these patients have been presented elsewhere [47].

Neonatal sepsis, birth asphyxia, malaria, neonatal jaundice and congenital malformations were the most common causes of admission and indication for transfer. This pattern is similar to studies done elsewhere except for the prominence of malaria in this study [17,22,29,45,48]. Prematurity and low birth weight were more prominent in some of the other studies [22,45]. Improving the treatment of malaria, impetigo and ophthalmia neonatorum in primary care will reduce the need for transfers. Since there was no paediatric surgeon at the regional hospital, some of the patients with congenital malformations had to be stabilised and referred on to one of two teaching hospitals which are 445 and 303 km away from UWRH. Thus, protocols for stabilisation and direct transfers need to be explored to save time [49]. It is unfortunate that one of the children who presented with bleeding after circumcision died since the condition can be prevented by ensuring that all babies, especially those born in the community, receive vitamin K and are treated with blood transfusion if necessary, though other causes of bleeding in the newborn need to be excluded [18,50].

The need to stabilise babies before and during transfer was highlighted by the study. Lack of appropriate transport systems puts these neonates at risk of poor outcome even when they are eventually admitted to hospital [28,29,30,48]. One way to address this is to shorten travel time by improving the quality of transport to minimise the effect of distance as studies have shown that neonates can be transferred safely across long distances by road [10,51]. Alternatively, distance to the closest facility can be reduced by increasing the number of admission facilities and bringing specialized care closer to communities [13]. Improving transportation seems more economically feasible but it requires logistics such as retrieval teams. These teams have been used in the UK and elsewhere since the 1960s to stabilise sick neonates before transfer across facilities, and it has improved outcomes [8,15,28,52,53]. Today they are common place in industrialised countries though they are manpower and resource intensive, requiring equipment such as transport incubators with ventilators and supporting structures, such as communication systems and specialized staff [28,34,52,53]. Although the cost of such a system may seem prohibitive for lower middle income countries like Ghana, modified forms could be used. Researchers in Ethiopia, studied women at risk of or having an obstetric complication transferred to hospital by a free ambulance system over a three month period, and found that it saved the lives of nine women and four newborns at a cost per year of life saved of $24.7 US dollars [54]. A similar scheme involving free ambulance services created in India has impacted neonatal transport in the public sector [20,45]. However more work is needed to improve quality and services in the private sector. Local and international standards have also been developed to improve neonatal care and transport [5,55,56]. However, these improvements must occur concurrently with health system strengthening of receiving facilities to make a significant impact [10,47,57].

Researchers in the Upper East region of Ghana, which shares a border with the Upper West Region also used a clinical audit programme involving five referral networks to strengthen the referral system in the region. The study led to improved referral rates of pregnant women and newborns, preference for and use of ambulances and facility-based vehicles, rather than taxis, and better communication between referring and destination hospitals. The use of escorts to facilitate referral was also found as well as better documentation through data collection, completion of registers and provision of feedback to referring hospitals [58]. A similar audit can be replicated in the Upper West Region incorporating mechanisms to sustain any positive changes. Studies from Canada have reported that some of these teams are nurse or technician-led, reinforcing the need to train different cadres of staff including nurse practitioners in neonatal medicine to support neonatal care from primary care to tertiary level. These nurses can support the work of physician assistants to stabilise, transfer and manage neonates in the community and form part of retrieval teams at tertiary centres. Some limitations were encountered. The choice of a descriptive cross-sectional design for this study rather than an analytic cross-sectional design has inherent limitations thus we were only able to provide clues about possible causes of mortality to prompt more rigorous studies [59,60]. We could not obtain data on all the patients admitted during the study period. There were fewer outborn patients transferred to SJH. Furthermore, the staff declined to participate after the extension of the study beyond June 2018. Financial and turnover time for the project prohibited further extension of the study. Thus the number of patients in this study was limited. This made it difficult for us to do a more detailed sub-analysis [13,14]. Furthermore, the fact that there were many variables of interest as against dependent variable, made it almost meaningless to do any co-linearity analysis with the view of doing logistic regression in the end. We felt that there is not enough variation distribution in the outcome variable to attempt to do the logistic regression. We did not assess how stable the patients were before transfer which could affect the clinical status on arrival. The diagnosis of sepsis was mainly clinical. This study provides a snapshot only of the patients who completed their referrals and does not include those who did not complete their referrals and long-term effects of poor transportation such as disability. Data on the timing or duration of travel was based on the judgment of the caregiver and may be subject to errors. A public health nurse from the Wa locality familiar with the terrain collected the data on most children so these errors could be minimised. Since the study was based only in the Upper West Region, the findings are limited to this area.

## 5. Conclusions

This study found that the median time spent at the first referral health facility by the patients who died was four times that of those who survived. In addition, a greater proportion of the patients who died travelled by public transport (bus) and arrived in an unstable condition. These finding suggests that there may have been delays initiating transfers or these babies may have needed more time to be stabilised before or during the transfer. Though the numbers were limited, the study highlights the need to improve the logistics of transfer to make it safe no matter the distance travelled. This requires upgrading skills for stabilising babies and treating certain conditions at first level referral facilities to avoid transfers or make it safer. It also requires provision of better transport, accompanied by skilled personnel and equipment as well as, a communication system or referral network that would facilitate transfers to reduce travel and waiting times. A larger case-control study on the subject as well as studies on the outcome of uncompleted referrals in this and similar settings is warranted.

## Figures and Tables

**Table 1:** Characteristics of outborn neonates referred to UWRH and SJH.

Demographic Characteristics	Outcome		
	Survived	Died	
	**n (%)**	**n (%)**	**χ^2^ (*p*-value)**
All participants	141 (92.2)	12 (7.8)	
Sex of neonate			
Female	56 (39.7)	9 (75.0)	5.634 (0.018)
Male	85 (60.3)	3 (25.0)	
Birth weight in grams			
<2500 g	15 (10.6)	6 (50.0)	14.468 (0.001)
≥2500 g	126 (89.4)	6 (50.0)	
Gestational age at birth			
<37 completed weeks	20 (14.2)	6 (50.0)	10.057 (0.002)
≥37 completed weeks	121 (85.8)	6 (50.5)	
Mother in attendance at NICU			
Mother in attendance	134 (95.0)	11 (91.7)	0.253 (0.615)
Mother did not attend *	7 (5.0)	1 (8.3)	
Accompanied to NICU by			
child’s father	66 (57.4)	2 (22.2)	5.721 (0.221)
Other family member	34 (29.6)	6 (66.7)	
Friend	13 (11.3.)	1 (11.1)	
Not indicated	2 (1.7)	-	
Time from onset of symptoms to arrival at hospital			
Within 24 h	37 (26.2)	2 (16.7)	0.534 (0.465)
More than 24 h	104 (73.8)	10 (83.3)	

* The mother was not in attendance in eight cases; two mothers died, three were on admission and the whereabouts of three were not indicated. UWRH: Upper West Regional Hospital; SJH: St Joseph’s Hospital, Jirapa; NICU: neonatal intensive care unit.

**Table 2:** Mode of transport to place of referral and sources of delays of outborn neonates during process of referral to UWRH and SJH.

	Survived	Died
**Transport Characteristics**	**N = 141**	**N = 12**
	n (%)	n (%)
Mode of Transport		
Walked	6 (4.3)	2 (16.7)
Taxi	50 (36.0)	2 (16.7)
Public transport (Bus)	40 (28.8)	5 (41.7)
Motor bike	39 (28.1)	2 (16.7)
Motor king (Tricycle)	2 (1.4)	1 (8.3)
Ambulance	1 (0.7)	-
* Unspecified	2 (1.4)	-
Self-reported experienced delay in reaching NICU		
Yes	29 (20.9)	2 (16.7)
No	110 (79.1)	10 (83.3)
Sources of delay among those reporting delay		
Bad road	19 (54.3)	
No available vehicle	8 (22.9)	2 (100.0)
The place was far	1 (2.9)	
Financial problem	5 (14.3)	
Other	2 (5.7)	
Time taken to be seen by doctor on arrival		
Less than 30 min	35 (24.8)	7 (58.3)
30–60 min	101 (71.6)	5 (41.6)
More than 60 min	5 (3.5)	-

* The unspecified transport includes one private transport.

**Table 3:** Condition and main diagnoses of outborn neonates on arrival at UWRH and SJH.

		Outcome	
		Survived	Died
**Clinical Characteristics**		**N = 141**	**N = 12**
		**n, %**	**n, %**
Temperature ranges (°C)			
Moderate to severe hypothermia	34.0–35.5	18 (12.8)	2 (16.7)
Mild hypothermia	35.6–36.4	49 (34.8)	6 (50.0)
Normal	36.5–37.5	15 (10.6)	3 (25.0)
High (low grade to moderate fever)	37.6–38.4	45 (31.9)	1 (8.3)
High (moderate fever to hyperpyrexia)	≥38.5	14 (9.9)	-
Heart rate (beats per min)			
Low	<110	36 (25.5)	5 (41.7)
Normal	111–160	99 (70.2)	5 (41.7)
High	>160	6 (4.3)	2 (16.7)
Respiratory rate (breaths/min)			
Low	<30	10 (7.1)	-
Normal	30–60	51 (36.4)	6 (50.0)
High	>60	79 (56.4)	6 (50.0)
General condition			
	Stable	35 (24.8)	1 (8.3)
	* Unstable	106 (75.2)	11 (91.7)
Main diagnosis			
	** Neonatal sepsis	26 (17.0)	2 (1.3)
	Asphyxia	14 (9.1)	2 (1.3)
	*** Malaria	13 (8.5)	
	Neonatal jaundice	13 (8.5)	
	Congenital malformation ****	13 (8.5)	
	Abdominal distension	11 (7.2)	1 (0.7)
	Prematurity	9 (5.9)	3 (1.9)
	Impetigo	9 (5.9)	
	Circumcision bleeding	8 (5.2)	1 (0.7)
	Ophthalmia neonatorum	8 (5.2)	
	Pneumonia	5 (3.2)	
	Low birth weight	4 (2.6)	2 (1.3)
	Birth injury	3 (1.9)	
	Gastroenteritis	3 (1.9)	
	Meconium aspiration	3 (1.9)	1 (0.7)
	Other *****	11 (7.5)	

* Neonates classified as unstable consist of those with at least one of the following signs: Twenty with moderate hypothermia (<35.6), 49 abnormal heart rate (<110 or >160) and 95 abnormal respiration (<30 or >60). Some had more than one sign. ** Neonatal sepsis includes eight cases of cord sepsis; *** malaria includes five cases of congenital malaria; **** The congenital malformations were preterm with deformity (one), hydrocephalus (three), exomphalos (three) and imperforate anus (six); birth injuries were preterm with fractured femur (one), trauma to the shoulder (one) and fracture of the left shoulder (one). ***** Other: Includes one patient each of anaemia, hypothermia, hypoglycaemia, baby not suckling well, caput succedaneum, neonatal seizure and macrosomic baby, as well as four cases with unspecified diagnosis.

## Abbreviations

IQR	Inter-quartile range
NICU	Neonatal intensive care unit
SD	Standard deviation
SDG	Sustainable development goals
SJH	St Joseph’s Hospital
UK	United Kingdom
UWRH	Upper West Regional Hospital

## References

[1] Lawn J.E., Cousens S., Zupan J. (2005). 4 million neonatal deaths: When? Where? Why?. Lancet.

[2] (2015). UNICEF, WHO, World Bank, UN-DESA Population Levels and Trends in Child Mortality: Report 2015: the UN Inter-Agency Group for Child Mortality Estimation.

[3] Bhutta Z.A., Das J.K., Bahl R., Lawn J.E., Salam R.A., Paul V.K., Walker N. (2014). Can available interventions end preventable deaths in mothers, newborn babies, and stillbirths, and at what cost?. Lancet.

[4] Vesel L., Manu A., Lohela T.J., Gabrysch S., Okyere E., ten Asbroek A.H., Kirkwood B.R. (2013). Quality of newborn care: a health facility assessment in rural Ghana using survey, vignette and surveillance data. BMJ Open.

[5] World Health Organization (2016). Standards for Improving Quality of Maternal and Newborn Care in Health Facilities.

[6] Målqvist M., Sohel N., Do T.T., Eriksson L., Persson L.-Å. (2010). Distance decay in delivery care utilisation associated with neonatal mortality. A case referent study in northern Vietnam. BMC Public Health.

[7] Bazzano A.N., Kirkwood B.R., Tawiah-Agyemang C., Owusu-Agyei S., Adongo P.B. (2008). Beyond symptom recognition: care-seeking for ill newborns in rural Ghana. Trop. Med. Int. Health.

[8] Storrs C.N., Taylor M.R. (1970). Transport of sick newborn babies. Br. Med. J..

[9] Nlend A.E.N., Zeudja C., Nsoa L. (2016). Transfert et transport des nouveau-nés en situation de détresse vitale à Yaoundé, Cameroun: analyse situationnelle dans un hôpital de référence. Pan. Afr. Med. J..

[10] Niermeyer S., Domek G. (2016). Neonatal transport in developing country settings: A systematic review. Pan American Health Organisation, Montevido. http://iris.paho.org/xmlui/handle/123456789/31317.

[11] Okwaraji Y.B., Edmond K.M. (2012). Proximity to health services and child survival in low-and middle-income countries: a systematic review and meta-analysis. BMJ Open.

[12] Schoeps A., Gabrysch S., Niamba L., Sié A., Becher H. (2011). The effect of distance to health-care facilities on childhood mortality in rural Burkina Faso. Am. J. Epidemiol..

[13] Karra M., Fink G., Canning D. (2017). Facility distance and child mortality: A multi-country study of health facility access, service utilization, and child health outcomes. Int. J. Epidemiol..

[14] King G., Zeng L. (2001). Logistic regression in rare events data. Political Analysis. Polit Anal..

[15] Cornette L. (2004). Contemporary neonatal transport: problems and solutions. Arch. Dis. Childhood Fetal Neonatal Ed..

[16] Chang Y.S. (2011). Regionalization of neonatal intensive care in Korea. Korean J. Pediatr..

[17] Rathod D., Adhisivam B., Bhat B.V. (2015). Transport of sick neonates to a tertiary care hospital, south India: condition at arrival and outcome. Trop Doct..

[18] Nalwadda C.K., Waiswa P., Kiguli J., Namazzi G., Namutamba S., Tomson G., Guwatudde D. (2013). High Compliance with Newborn Community-to-Facility Referral in Eastern Uganda: An Opportunity to Improve Newborn Survival. PLoS ONE.

[19] Kozuki N., Guenther T., Vaz L., Moran A., Soofi S.B., Kayemba C.N., Doherty T. (2015). A systematic review of community-to-facility neonatal referral completion rates in Africa and Asia. BMC Public Health.

[20] Roy M.P., Gupta R., Sehgal R. (2016). Neonatal transport in India: From public health perspective. Med J. Dr DY Patil Univ..

[21] Hedstrom A., Ryman T., Otai C., Nyonyintono J., McAdams R.M., Lester D., Batra M. (2014). Demographics, clinical characteristics and neonatal outcomes in a rural Ugandan NICU. BMC Pregnancy Childbirth.

[22] Elwan A. (2009). Mortality Among Outborn Versus Inborn Neonates: A Retrospective Comparative Study. Med. J. Cairo Univ..

[23] Ghana Statistical Service (2013). 2010 Population & Housing Census: National Analytical Report.

[24] Ghana Health Service (2019). Upper West Region—Upper West Regional Directorate. Upper West Region. http://www.ghanahealthservice.org/rhdcategory.php?ghsrid=3&cid=39.

[25] Fleming S., Thompson M., Stevens R., Heneghan C., Plüddemann A., Maconochie I., Mant D. (2011). Normal ranges of heart rate and respiratory rate in children from birth to 18 years of age: a systematic review of observational studies. Lancet.

[26] World Health Organization (1993). Thermal Control of the Newborn: A Practical Guide.

[27] Mears M., Chalmers S. (2005). Neonatal pre-transport stabilisation–caring for infants the STABLE way. Infant.

[28] Lee K.-S. (2019). Neonatal transport metrics and quality improvement in a regional transport service. Transl. Pediatr..

[29] Aggarwal K.C., Gupta R., Sharma S., Sehgal R., Roy M.P. (2015). Mortality in newborns referred to tertiary hospital: An introspection. J. Family Med. Prim. Care.

[30] Abdulraheem M.A., Tongo O.O., Orimadegun A.E., Akinbami O.F. (2016). Neonatal transport practices in Ibadan, Nigeria. Pan Afr. Med J..

[31] Baidya M., Shirin M., Saha L.C. (2017). Transport Factors Affecting the Outcome of Referred Neonates Admitted in A Tertiary Care Hospital. Bangladesh J. Child Health.

[32] Dey S.K., Sharker S., Jahan I., Moni S.C., Shabuj K.H., Chisti M.J., Shahidullah M. (2017). Neonatal Transport—Experience of a Tertiary Care Hospital of Bangladesh. Mymensingh Med. J..

[33] Whyte H.E., Jefferies A.L. (2015). The interfacility transport of critically ill newborns. None.

[34] Nimbalkar S., Patel H., Dongara A., Patel D.V., Bansal S. (2014). Usage of EMBRACETM in Gujarat, India: Survey of Paediatricians. Adv. Prev. Med..

[35] Agourram B., Bach V., Tourneux P., Krim G., Delanaud S., Libert J.-P. (2010). Why wrapping premature neonates to prevent hypothermia can predispose to overheating. J. Appl. Physiol..

[36] Bowman E.D., Roy R.N.D. (1997). Control of temperature during newborn transport: an old problem with new difficulties. J. Paediatr. Child Health.

[37] Advanced Life Support Group (ALSG) (2011). Advanced Paediatric Life Support: The Practical Approach.

[38] Dawson J.A., Kamlin C.O.F., Wong C., Te Pas A.B., Vento M., Cole T.J., Morley C.J. (2010). Changes in heart rate in the first minutes after birth. Arch. Dis. Childhood Fetal Neonatal Ed..

[39] Chong S.L., Ong G.Y.K., Chin W.Y.W., Chua J.M., Nair P., Ong A.S.Z., Maconochie I. (2018). A retrospective review of vital signs and clinical outcomes of febrile infants younger than 3 months old presenting to the emergency department. PLoS ONE.

[40] Griffin M.P., Lake D.E., O’Shea T.M., Moorman J.R. (2007). Heart rate characteristics and clinical signs in neonatal sepsis. Pediatric Res..

[41] Henry S., Trotman H. (2017). Challenges in neonatal transport in Jamaica: A resource-limited setting. J. Trop. Pediatrics.

[42] Narli N., Kırımi E., Uslu S. (2018). Turkish Neonatal Society guideline on the safe transport of newborn. Turk Pediatri Ars..

[43] Jajoo M., Kumar D., Dabas V., Mohta A. (2017). Neonatal transport: The long drive has not even begun. Indian J. Community Med..

[44] Ashokcoomar P., Naidoo R. (2016). An analysis of inter-healthcare facility transfer of neonates within the eThekwini Health District of KwaZulu-Natal, South Africa. South Afr. Med J..

[45] Punitha P., Kumaravel K.S., Pugalendhiraja K.V. (2016). A Study on The Current Status of Neonatal Transport to A Special Newborn Care Unit. Stanley Med J..

[46] Sumankuuro J., Crockett J., Wang S. (2018). Perceived barriers to maternal and newborn health services delivery: a qualitative study of health workers and community members in low and middle-income settings. BMJ Open.

[47] Tette E., Nuertey B.D., Azusong E.A., Gandau N.B. (2020). The Profile, Health Seeking Behavior, Referral Patterns, and Outcome of Outborn Neonates Admitted to a District and Regional Hospital in the Upper West Region of Ghana: A Cross-Sectional Study. Children.

[48] Narang M., Kaushik J.S., Sharma A.K., Faridi M.M.A. (2013). Predictors of mortality among the neonates transported to referral centre in Delhi, India. Indian J. Public Health.

[49] Arul G.S., Spicer R.D. (1998). Where should paediatric surgery be performed?. Archives Dis. Child..

[50] El Hasbaoui B., Karboubi L., Benjelloun B.S. (2017). Newborn haemorrhagic disorders: About 30 cases. Pan Afr. Med. J..

[51] Kumar P.P., Kumar C.D., Shaik F.A., Ghanta S.B., Venkatalakshmi A. (2010). Prolonged neonatal interhospital transport on road: relevance for developing countries. Indian J. Pediatrics.

[52] Britto J., Nadel S., Maconochie I., Levin M., Habibi P. (1995). Morbidity and severity of illness during interhospital transfer: impact of a specialised paediatric retrieval team. BMJ.

[53] Droogh J.M., Smit M., Absalom A.R., Ligtenberg J.J., Zijlstra J.G. (2015). Transferring the critically ill patient: are we there yet?. Critical Care.

[54] Accorsi S., Somigliana E., Solomon H., Ademe T., Woldegebriel J., Almaz B., Seifu A. (2017). Cost-effectiveness of an ambulance-based referral system for emergency obstetrical and neonatal care in rural Ethiopia. BMC Pregnancy Childbirth.

[55] Ghana Health Service (2019). Ghana National Newborn Health Strategy and Action Plan 2019-2023.

[56] Stroud M.H., Trautman M.S., Meyer K., Moss M.M., Schwartz H.P., Bigham M.T., Meyer M.T. (2013). Pediatric and neonatal interfacility transport: results from a national consensus conference. Pediatrics.

[57] Tette E.M., Neizer M., Nyarko M.Y., Sifah E.K., Nartey E.T., Donkor E.S. (2016). Changing patterns of disease and mortality at the Children’s Hospital, Accra: are infections rising?. PLoS ONE.

[58] Awoonor-Williams J.K., Bailey P.E., Yeji F., Adongo A.E., Baffoe P., Williams A., Mercer S. (2015). Conducting an audit to improve the facilitation of emergency maternal and newborn referral in northern Ghana. Global Public Health.

[59] Grimes D.A., Schulz K.F. (2002). Descriptive studies: what they can and cannot do. Lancet.

[60] Aggarwal R., Ranganathan P. (2019). Study designs: Part 2—Descriptive studies. Perspect. Clin. Res..

